# Diagnosis and Management of Obesity Hypoventilation Syndrome during Labor

**DOI:** 10.1155/2021/8096212

**Published:** 2021-08-25

**Authors:** Brandon M. Togioka, Sarah S. McConville, Rachael M Penchoen-Lind, Katie J. Schenning

**Affiliations:** ^1^Department of Anesthesiology and Perioperative Medicine, Oregon Health & Science University, Portland, OR, USA; ^2^Department of Obstetrics and Gynecology, Oregon Health & Science University, Portland, OR, USA; ^3^Department of Neurology, Oregon Health & Science University, Portland, OR, USA

## Abstract

Obesity hypoventilation syndrome (OHS) is a disorder in which patients with a body mass index ≥30 kg/m^2^ develop awake hypercapnia with a partial pressure of carbon dioxide ≥45 mm Hg, in the absence of other diseases that may produce alveolar hypoventilation. Additional clinical features include sleep disordered breathing, restrictive lung disease, polycythemia, hypoxemia, and an increased serum bicarbonate concentration (≥27 mEq/L). Anesthesia providers should be familiar with OHS because it is often undiagnosed, it is associated with a higher mortality rate than obstructive sleep apnea, and it is projected to increase in prevalence along with the obesity epidemic. In this case, a 33-year-old obese woman with presumed OHS developed respiratory acidosis during induction of labor. Continuous positive airway pressure treatment was initiated, but the patient continued to have hypercapnia. A cesarean delivery was recommended. The patient had baseline orthopnea due to her body habitus; thus, despite adequate labor analgesia, a cesarean delivery was completed with general endotracheal anesthesia. We believe this patient had OHS despite a serum bicarbonate <27 mEq/L, a partial pressure of oxygen >70 mm Hg, and a hemoglobin <16 g/dL, which would typically rule out OHS. Pregnant women experience a decrease in serum bicarbonate concentration due to progesterone-mediated hyperventilation, an increase in arterial oxygenation from increased minute ventilation and higher cardiac output, and a decrease in hemoglobin due to the physiologic anemia of pregnancy. Thus, OHS may be defined differently in pregnant than in non-pregnant patients.

## 1. Introduction

Obesity hypoventilation syndrome (OHS) is a disorder in which patients with body mass index (BMI) >30 kg/m^2^ develop awake hypercapnia with partial pressure of carbon dioxide (P_a_CO_2_) >45 mm Hg, in the absence of other diseases that may produce alveolar hypoventilation [1]. Additional clinical features include restrictive lung disease and polycythemia (hemoglobin >16 g/dL) [1]. Obstructive sleep apnea (OSA) is present in 90% of individuals with OHS [2]. Serum bicarbonate has been used to screen for OHS, with a level <27 mEq/L decreasing the probability of the disease [3, 4]. The prevalence of OHS has been estimated to be between 0.15 and 0.4% of the world's adult population [5, 6]. The prevalence of OHS is expected to increase along with the obesity epidemic [7, 8]. Patients with OHS are high healthcare utilizers and they generate higher physician fees [9]. OHS is associated with a higher mortality rate than OSA [10] and obesity without hypoventilation [11]; thus, it is critical that anesthesia providers learn how to diagnose OHS [12]. However, OHS may be defined differently in pregnant than in non-pregnant patients.

Our first objective is to highlight OHS as a risk factor for respiratory acidosis during labor. Our second objective is to illustrate that the established tests for OHS lack utility in the obstetric population. Health Insurance Portability and Accountability Act written authorization for publication of this case report was obtained from the patient. This paper adheres to the CARE guidelines [13].

## 2. Case Presentation

A 33-year-old woman with a BMI of 58.6 kg/m^2^, type 2 diabetes (hemoglobin A1C 6.6%) with nephropathy (baseline creatinine 1.5–1.7 mg/dL), and a prior myocardial infarction was admitted to the hospital for sustained blood pressures >200/100 mm Hg and a posterior cranial headache. Her hypertensive emergency was attributed to 10 days without anti-hypertension medications; she was prescribed 10 mg once daily oral amlodipine and 50 mg once daily oral hydrochlorothiazide. During that admission, the patient's serum bicarbonate ranged between 27 and 33 mmol/L, consistent with OHS.

Two years later, with a 35-week gestational pregnancy, the patient was again admitted to the hospital for hypertension and a headache. A diagnosis of preeclampsia superimposed upon chronic hypertension (baseline systolic blood pressure of 130–145 mm Hg) was made after blood pressures of 157/74 mm Hg (automated oscillometric technique) and 164/92 mm Hg (manual auscultatory technique) were recorded four hours apart. The patient's chronic hypertension was treated with 200 mg twice daily oral labetalol and 10 mg once daily oral amlodipine. She reported no missed medication doses.

The patient's obstetric history included one prior full-term vaginal delivery. Her admission non-stress test was categorized as reactive with a baseline heart rate of 125 beats per minute, moderate variability, and qualifying accelerations present. Her medical history additionally included mild intermittent asthma (no wheezing or coughing during pregnancy), anemia (hemoglobin 11.1 g/dL), and moderate likely extrapulmonary restrictive lung disease (2nd trimester spirometry FEV1 68%, FVC 65%, total lung capacity 78%) [14]. She was confirmed to be euthyroid with 2nd trimester testing (thyroid-stimulating hormone 1.78 uIU/ml).

Physical examination revealed a neck circumference >40 cm, Mallampati class IV airway, and clear lungs. The patient was in a labor bed that had been modified to a sitting position due to prepregnancy chest discomfort and subjective shortness of breath (oxygen saturation 98% on room air) that occurred when the patient was supine. A home sleep study was completed 4 days prior to admission, due to a history of snoring and chronic fatigue, but the results were not finalized at the time of admission.

Despite increasing oral labetalol to 600 mg twice daily, the patient's blood pressure climbed to 167/90 mm Hg and her creatinine increased to 1.5 mg/dL. Cervical ripening, lactated ringer's infusion, and magnesium (4 gram load followed by 1 gram/hour) were initiated. The patient described worsening shortness of breath and was given a non-rebreather mask (NRB) delivering oxygen at 10 liters per minute. Her oxygen saturation remained low at 92%. Albuterol (2.5 mg of nebulizer solution) was empirically administered despite a lack of wheezing on auscultation. Albuterol did not improve her oxygen saturation or shortness of breath. Pulmonology was consulted and continuous positive airway pressure (CPAP) was prescribed for “likely OSA,” but the patient declined it.

The next day, the patient was taken to the operating room (given high fetal station) for epidural placement, artificial rupture of membranes, and placement of an internal fetal monitor followed by misoprostol administration and initiation of oxytocin infusion. An epidural was placed at the L3/4 vertebral interspace, and 7.5 mL of 0.055% bupivacaine with 1 mcg/mL sufentanil solution was administered, causing an upper level of loss of pinprick sensation to the T10 dermatome. Epidural analgesia was maintained using the CADD-Solis Infusion System version 3.0 (Smiths Medical, Minneapolis, MN) with a 7 mL programmed bolus every 45 minutes and a 5 mL patient-controlled bolus every 10 minutes. The patient reported no pain and maintained modified Bromage grade 0 (free movement of legs and feet) motor function throughout her induction [15].

**Figure 1:**
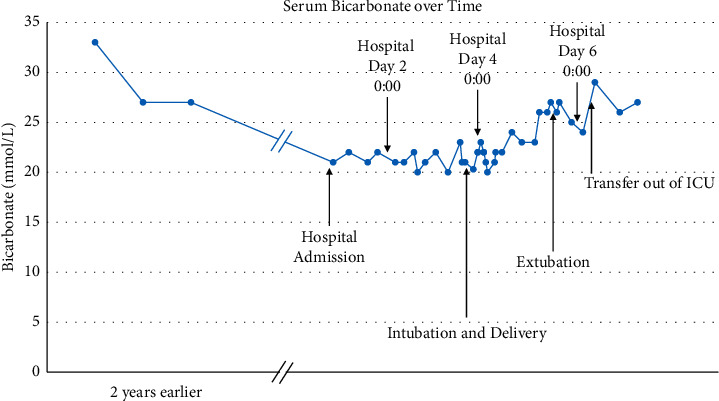
Serum bicarbonate changes from preconception to postpartum. ICU, intensive care unit; L, liter; mmol, millimole.

Sixteen hours after epidural placement, the patient was not in active labor and she complained of difficulty in breathing. A chest radiograph revealed an elevated diaphragm without consolidation or pulmonary edema. A formal transthoracic echocardiogram revealed severe concentric left ventricular hypertrophy with an ejection fraction of 65%, a severely dilated left atrium, and a right ventricle with normal size and function. There was no tricuspid regurgitation, and thus pulmonary artery pressure could not be assessed. An arterial blood gas (ABG) and metabolic panel revealed normochloremic acidosis (pH 7.27, bicarbonate 21 mmol/L, and chloride 106 mmol/L), hypercarbia (P_a_CO_2_ 47 mmHg), hypoxia (P_a_O_2_ 96 mmHg on NRB delivering oxygen at 10 LPM), and hypermagnesemia (4.9 mg/dL) (Table 1 and Figure 1). For comparison, a normal term pregnancy ABG is pH 7.43, P_a_CO_2_ 30.4 mmHg, P_a_O_2_ 101.8 mmHg on 21% oxygen, and bicarbonate 21.7 mmol/L [8]. The patient's hypoxia and hypercarbia were attributed to increased carbon dioxide production associated with induction of labor, myasthenia caused by hypermagnesemia, and respiratory muscle fatigue. The patient agreed to use CPAP (10 cm H_2_O, 100% oxygen), but an ABG one hour later did not show an improvement in respiratory acidosis: pH 7.26, P_a_CO_2_ 49 mmHg, P_a_O_2_ 124 mmHg, and bicarbonate 21 mmol/L (Table 1). A cesarean delivery was recommended for maternal respiratory compromise, but the patient declined.

Seven hours after initiating CPAP treatment, cervical dilation was unchanged and the patient agreed to cesarean delivery. Despite adequate epidural analgesia, the patient required general anesthesia with endotracheal intubation for baseline orthopnea due to her body habitus. She was administered 30 ml oral sodium citrate, induced with propofol, paralyzed with rocuronium, and intubated with a video laryngoscope in the reverse-Trendelenburg position. Skin incision to delivery took 20 minutes. Significant adipose tissue made surgical dissection to the lower uterine segment and fetal head extraction difficult. Although the hysterotomy had to be extended resulting in a 2000 mL blood loss, hemodynamic stability was maintained with administration of 3000 mL lactated ringer's solution, and blood transfusion was not administered. Apgar scores were 1 (1 minute) and 4 (5 minutes), and the neonate was intubated. The neonate was extubated the next day and discharged home in good health. After surgery, the patient was transported intubated to the intensive care unit (ICU).

The patient was afebrile, but remained mechanically ventilated due to preeclampsia-related acute on chronic kidney injury (creatinine 2.82 mg/dL and potassium 6.7 mmol/L). Hyperkalemia was successfully treated with a 20 mg/hour intravenous furosemide infusion, renal function improved without hemodialysis, and the patient was extubated on postdelivery day 2. One day after extubation, the patient's serum bicarbonate was 29 mmol/L (Figure 1).

The remainder of the patient's hospitalization was unremarkable. On the day of discharge, the patient's home sleep study was finalized revealing an apnea hypopnea index of 46.8 events/hour, an obstructive apnea index of 18.2 events/hour, a 90% mean oxygen saturation, and a 68% oxygen saturation nadir. The patient was discharged home with an auto-CPAP machine set to a range of 5–15 cm H_2_O.

Due to COVID-19 precautionary measures, the patient had a telephone appointment with the sleep disorders clinic 5 months after discharge. The patient reported rare CPAP use, due to not liking the face mask fit. A plan was made to complete a positive airway pressure titration study to determine if bilevel positive airway pressure may reduce the number of obstructive or hypoxic events the patient experienced at night. No date has been set for this appointment. A phone call one year after delivery confirmed that the patient and infant are doing well and CPAP use has remained inconsistent.

## 3. Discussion

Although the prevalence of OHS is increasing in the obstetric population and OHS is known to increase risk for perioperative morbidity and mortality [16], there are limited reports of obstetric patients with OHS. We found only one case report describing OHS during pregnancy, in which a parturient with obesity-related hypoventilation and hypercarbia had cardiac arrest after developing severe hypokalemia due to hyperemesis gravidarum [17]. By contrast, increased attention is being paid to the diagnosis and treatment of OSA during pregnancy. OSA is common in pregnant women, the incidence increases with higher gestational age [18] and BMI [19], and it is associated with preeclampsia and gestational diabetes [18, 19]. While the two diseases share clinical manifestations and pathogenesis, it is important to distinguish between them by assessing for awake hypercapnia (P_a_CO_2_ >45 mm Hg) because patients with OHS have poorer prognoses and higher mortality rates than patients with OSA [9, 20].

In this case, a patient with presumed OHS developed acute respiratory acidosis as a result of multiple physiologic challenges during induction of labor, including increased carbon dioxide production, a blunted respiratory response to hypercarbia, and respiratory muscle fatigue. CPAP therapy was initiated, but the patient continued to have hypercapnia. It was determined that cesarean delivery may help the patient regain acid-base homeostasis. The patient had baseline orthopnea due to her body habitus; thus, despite adequate labor analgesia, a cesarean delivery was completed with general endotracheal anesthesia.

Appropriate screening and identification of OHS in this patient may have facilitated vaginal delivery with neuraxial labor analgesia. In the short term, positive pressure ventilation (PPV) can reduce chest discomfort and upper airway obstruction for patients in the supine position [21]. Long-term PPV has been shown to normalize P_a_CO_2_, improve oxygenation, reduce daytime sleepiness, improve functional status, improve pulmonary mechanics, stimulate central respiratory response to hypercarbia, and reduce mortality [22–25]. Therefore, a formal diagnosis of OHS earlier in pregnancy, conditioning to PPV, and use of PPV during induction of labor may have prevented the need for mechanical ventilation.

The established differential diagnosis for acute respiratory acidosis during labor includes asthma exacerbation, pneumonia, cardiogenic and non-cardiogenic pulmonary edema, hypermagnesemia, and pulmonary embolism. In the present case, these diagnoses were deemed less likely than respiratory acidosis related to OHS: asthma (no wheezing and no response to albuterol), pneumonia (no fever and clear chest radiograph), pulmonary edema (clear chest radiograph), hypermagnesemia (lack of hyporeflexia and a peak magnesium concentration of 4.9 mg/dL, whereas levels >15 mg/dL are typically associated with respiratory arrest), and pulmonary embolism (no chest pain, hemoptysis, or cough).

The preponderance of evidence supports a diagnosis of OHS, and that it led to the patient's acute respiratory acidosis. The patient possessed many of the criteria used to diagnose OHS: high BMI (58.6 kg/m^2^), restrictive lung disease, diaphragm elevation on chest radiograph, severe OSA (apnea hypopnea index 46.8 events/hour), and daytime awake hypercapnia (P_a_CO_2_ >45 mm Hg) [1]. Although P_a_CO_2_ >45 mm Hg is not impressive outside of pregnancy, during pregnancy it is consistent with significant respiratory acidosis, as normal term pregnancy P_a_CO_2_ is 30 mm Hg [8].

The diagnostic criteria for OHS that the patient did not possess (serum bicarbonate ≥27 mEq/L, P_a_O_2_ <70 mm Hg, and hemoglobin >16 g/dL) can be explained by physiologic changes associated with pregnancy and labor (Table 2).

Serum bicarbonate concentration ≥27 mEq/L was found to predict hypercapnia on arterial blood gas measurement with 92% sensitivity [26]. This prompted the recommendation that clinicians use this easily obtainable lab value as an initial screening test for OHS [26]. In this case, the patient's serum bicarbonate was ≥27 mEq/L before becoming pregnant and after delivery; however, her serum bicarbonate was 20–23 mEq/L during her induction of labor (Figure 1). Serum bicarbonate decreases during pregnancy due to metabolic compensation for progesterone-mediated respiratory alkalosis [8]. Serum bicarbonate may further decrease during labor when pain causes hyperventilation. Therefore, the 27 mEq/L threshold for serum bicarbonate may have lower sensitivity in pregnant women. Consequently, we suggest a measurement of P_a_CO_2_ from an ABG in pregnant women, in whom suspicion for OHS is high.

The patient was not hypoxemic during the majority of her peripartum course (Table 1). P_a_O_2_ increases 10 mmHg during pregnancy due to increased minute ventilation, lower P_a_CO_2_ (which increases P_a_O_2_ as calculated by the alveolar gas equation), and a smaller drop in venous oxygen concentration due to increased cardiac output [8]. Hence, physiologic changes associated with pregnancy decrease the likelihood that parturients will be hypoxic, which would increase the number of false negatives and decrease the sensitivity of P_a_O_2_ <70 mm Hg for diagnosing OHS.

Though the patient's hemoglobin was never >16 g/dL, the patient's hemoglobin dropped from 14.6 g/dL at 8 weeks gestational age to 10.9–11.3 g/dL in the third trimester. The patient's hemoglobin was not measured prior to pregnancy. The body prepares for blood loss during delivery by increasing plasma volume more than red cell mass, known as physiologic anemia of pregnancy [8]. Thus, polycythemia is rare during pregnancy and may not be useful as a diagnostic test for OHS during pregnancy.

In conclusion, the diagnosis of OHS in pregnancy is not straightforward. The diagnostic criteria for OHS in non-pregnant patients have low sensitivity in patients with physiologic changes associated with pregnancy and labor. We have outlined the diagnostic findings associated with OHS in non-pregnant patients, the associated physiologic changes of pregnancy, and the impact of pregnancy on the sensitivity of those diagnostic criteria (Table 2). We described a case of hypercapnic respiratory failure in a pregnant woman with presumed OHS, who required general endotracheal anesthesia for cesarean delivery—highlighting the need for early diagnosis and treatment with PPV in these patients.

## Figures and Tables

**Table 1:** Arterial blood gas values from preconception to postpartum.

Time point, relative to day of admission	Partial pressure of carbon dioxide (mm Hg)	Partial pressure of oxygen (mm Hg)	Serum bicarbonate concentration (mmol/L)	Oxygen delivery type	Fraction of inspired oxygen (%) or delivery of oxygen (L/min)
2 years prior to admission	n/a	n/a	27–33	Natural airway	21%
Hospital day 1, time 23 : 11	n/a	n/a	21	Natural airway	21%
Hospital day 2, time 7 : 01	n/a	n/a	22	Natural airway	21%
Hospital day 2, time 16 : 27	n/a	n/a	21	Natural airway	21%
Hospital day 2, time 21 : 23	n/a	n/a	22	Natural airway	21%
Hospital day 3, time 6 : 15	n/a	n/a	21	Natural airway	21%
Hospital day 3, time 10 : 39	n/a	n/a	21	Natural airway	21%
Hospital day 3, time 15 : 38	n/a	n/a	22	Natural airway	21%
Hospital day 3, time 17 : 26	n/a	n/a	20	Natural airway	21%
Hospital day 3, time 21 : 14	n/a	n/a	21	Natural airway	21%
Hospital day 4, time 2 : 30	n/a	n/a	22	Natural airway	21%
Hospital day 4, time 8 : 39	n/a	n/a	20	Natural airway	21%
Hospital day 4, time 14 : 44	n/a	n/a	23	NRM	10 l/min
Hospital day 4, time 15 : 40	47	96	21	NRM	10 l/min
Hospital day 4, time 17 : 13	49	124	21	CPAP	100%
Hospital day 4, time 21 : 20	48	125	20	ETT	100%
Hospital day 4, time 23 : 27	n/a	n/a	22	ETT	40%
Hospital day 4, time 23 : 39	68	90	22	ETT	40%
Hospital day 5, time 1 : 05	61	63	23	ETT	40%
Hospital day 5, time 2 : 32	n/a	n/a	22	ETT	80%
Hospital day 5, time 2 : 33	54	94	22	ETT	80%
Hospital day 5, time 3 : 33	47	78	21	ETT	50%
Hospital day 5, time 4 : 22	n/a	n/a	20	ETT	50%
Hospital day 5, time 7 : 58	n/a	n/a	21	ETT	50%
Hospital day 5, time 8 : 28	43	83	22	ETT	50%
Hospital day 5, time 11 : 40	n/a	n/a	22	ETT	50%
Hospital day 5, time 16 : 39	n/a	n/a	24	ETT	50%
Hospital day 5, time 21 : 37	41	90	21	ETT	50%
Hospital day 6, time 4 : 05	n/a	n/a	23	ETT	40%
Hospital day 6, time 6 : 24	42	93	26	ETT	40%
Hospital day 6, time 10 : 20	n/a	n/a	26	ETT	40%
Hospital day 6, time 12 : 05	42	101	27	ETT	40%
Hospital day 6, time 15 : 05	n/a	n/a	26	CPAP	25%
Hospital day 6, time 16 : 21	37	69	27	CPAP	25%
Hospital day 6, time 22 : 29	n/a	n/a	25	Natural airway	21%
Hospital day 7, time 4 : 10	n/a	n/a	24	CPAP	25%
Hospital day 7, time 10 : 11	n/a	n/a	29	Natural airway	21%
Hospital day 7, time 22 : 36	n/a	n/a	26	CPAP	25%
Hospital day 8, time 7 : 32	n/a	n/a	27	CPAP	25%

CPAP, continuous positive airway pressure; ETT, endotracheal tube; L, liter; min, minute; mm Hg, millimeter of mercury; mmol, millimole; n/a, not available; NRM, non-rebreather mask.

**Table 2:** Impact of physiologic changes of pregnancy on diagnostic findings associated with obesity hypoventilation syndrome.

Diagnostic finding	Pertinent physiologic changes of pregnancy	Impact of pregnancy on likelihood of meeting diagnostic criteria
BMI >30 kg/m^2^	Healthy weight gain in pregnancy, which can be up to 40 pounds	Increased likelihood

Awake hypercapnia, P_a_CO_2_ >45 mm Hg	Progesterone-mediated 40–50% increase in minute ventilation causing respiratory alkalosis	Decreased likelihood

Awake hypoxemia, P_a_O_2_ <70 mm Hg	P_a_O_2_ increases 10 mm Hg during normal pregnancy due to hyperventilation, a lower P_a_CO_2_, and a smaller drop in venous oxygen concentration due to increased cardiac output	Decreased likelihood

Elevated serum bicarbonate ≥27 mEq/L	Respiratory alkalosis with renal compensation, which decreases plasma bicarbonate	Decreased likelihood

Polycythemia, hemoglobin >16 g/dL	Physiologic anemia of pregnancy, 30% increase in plasma volume with lower increase in red cell mass	Decreased likelihood

Restrictive lung pattern on PFT, FVC <80% predicted	Lung restriction due to enlarged uterus	Increased likelihood

Elevation diaphragm on chest X-ray	Elevation of diaphragm due to enlarged uterus	Increased likelihood

BMI, body mass index; FVC, forced vital capacity; kg, kilogram; L, liter; m, meter; mEq, milliequivalent; mm Hg, millimeter of mercury; P_a_CO_2_, partial pressure of carbon dioxide; P_a_O_2_, partial pressure of oxygen; PFT, pulmonary function test.

## Data Availability

The data used to support the findings of this study are available from the corresponding author upon request.
